# Case Study: A Dementia Strategy for Canada: Together We Aspire

**DOI:** 10.1002/alz70860_110733

**Published:** 2025-12-23

**Authors:** Franca Gatto

**Affiliations:** ^1^ Public Health Agency of Canada, Ottawa, ON, Canada

## Abstract

In 2019, Canada issued its first national dementia strategy. It has three overarching national objectives: prevent dementia, advance therapies and find a cure, and improve the quality of life of people living with dementia and caregivers. This presentation will provide an overview of the scope of the strategy and share highlights of progress made toward those objectives.

